# Steering generative models for protein design: Aligning and conditioning strategies

**DOI:** 10.1016/j.sbi.2026.103250

**Published:** 2026-06

**Authors:** Filippo Stocco, Michele Garibbo, Noelia Ferruz

**Affiliations:** 1 Centre for Genomic Regulation, The Barcelona Institute of Science and Technology, Dr Aiguader 88, Barcelona 08003, Spain; 2 Universitat Pompeu Fabra (UPF), Barcelona, Spain

## Abstract

Generative artificial intelligence models learn probability distributions from data and produce novel samples that capture the salient properties of their training sets. Proteins are particularly attractive for such approaches given their abundant data and the versatility of their representations, ranging from sequences to structures and functions. This versatility has motivated the rapid development of generative models for protein design, enabling the generation of functional proteins and enzymes with unprecedented success. However, because these models mirror their training distribution, they tend to sample from its most probable modes, while low-probability regions, often encoding valuable properties, remain underexplored. To address this challenge, recent work has proposed strategies for steering generative models toward user-specified properties. In this review, we survey and categorize these strategies, distinguishing approaches that modify model parameters, such as reinforcement learning or supervised fine-tuning, from those that keep the model’s parameters fixed, including conditional generation, retrieval-augmented strategies, Bayesian guidance, and tailored sampling methods. Together, these developments are beginning to enable the steering of generative models toward proteins with desired properties.

## Highlights

• Generative AI models have reshaped the field of protein design, enabling the generation of functional proteins and enzymes with unprecedented success.
• However, these models tend to mirror their training distribution, which does not necessarily align with the objectives required for protein engineering.
• Recent work proposes strategies for steering generative models toward user-defined properties, and we categorize these efforts into parameter-updating alignment and parameter-fixed steering.
• Parameter-updating Alignment approaches modify the model weights, either through fine-tuning or by optimizing with external oracles via rewards (reinforcement learning).
• Parameter-fixed steering approaches guide the model at inference time through conditioning, retrieval, guidance, or sampling strategies.

## Introduction

**Figure 1:**
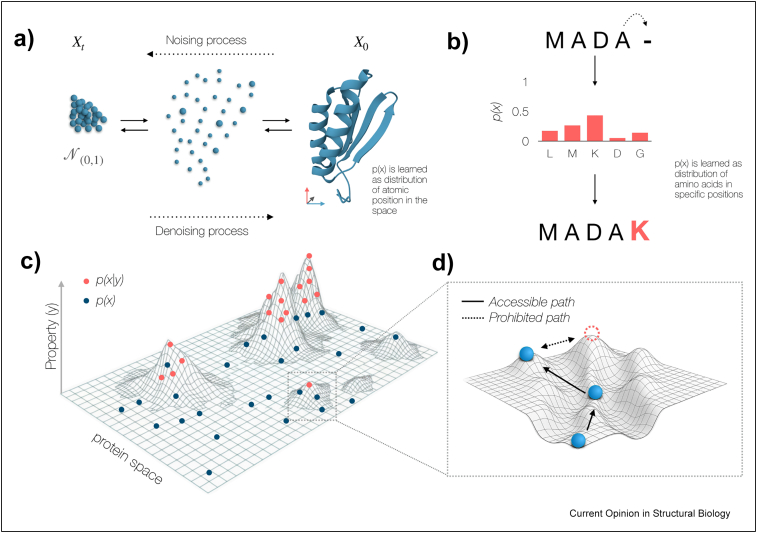
**Generative modeling approaches and schematic illustrations of protein fitness landscapes.** (**a**) *Diffusion-based generative models.* Protein structures are represented by the distribution *p*(*x*) of atomic coordinates in three-dimensional space. During training, true atomic coordinates *X*_0_ are progressively corrupted by Gaussian noise until reaching a normal distribution N(0,1). At inference, the model reverses this process, iteratively denoising random noise to reconstruct a protein structure. (**b**) *Sequence-based generative models.* Here, *p*(*x*) denotes the distribution of amino acids across sequence positions. Training can proceed via masked-token prediction, in which the model infers the identity of masked residues, or via autoregressive next-token prediction, where sequences are generated one residue at a time. (**c**) *Protein fitness landscape.* Schematic illustration of the relationship between the data distribution *p*(*x*) (blue points), typically learned in an unsupervised manner, and the conditional distribution *p*(*y*|*x*) (red points), which focuses sampling on regions associated with high fitness. (**d**) *Evolutionary accessibility.* Evolution explores the fitness landscape through local, incremental mutations that can reach only contiguous high-fitness regions (solid paths), whereas transitions across fitness valleys (dashed paths) are inaccessible. In contrast, generative protein design models can, in principle, traverse the landscape in a less constrained manner, directly sampling from otherwise evolutionarily inaccessible regions.

Learning to generate samples from complex probability distributions lies at the core of modern generative modeling. In the context of proteins, the availability of large datasets has catalyzed the rapid development of powerful generative models (GMs) for protein design. Some of the most adopted architectures are diffusion models, which reconstruct atomic coordinates by reversing a noise-adding process (Figure 1a) [1, 2, 3], and protein language models (pLMs), trained either on masked sequence reconstruction (e.g. Evolutionary Scale Modeling (ESM) [4] or the Multiple Sequence Alignment (MSA) Transformer [5]) or on autoregressive next-token prediction (e.g. ProtGPT2 [6], ProGen2-3 [7], and Evo-1/2 [8,9]) (Figure 1b). In recent years, such models have enabled remarkable achievements, including the design of binders [10], ligand-binding receptors [11], and *de novo* enzymes [12] on unprecedented timescales. For broader overviews of generative artificial intelligence for protein design, we refer the reader to recent reviews [13, 14, 15].

Trained in a large corpus of natural sequences, structures, and functional annotations (e.g. Refs. [7,16, 17, 18]), GMs can explore vast regions of the protein sequence–structure landscape (e.g. Refs. [7,19, 20, 21]). However, protein engineering often targets exceptional properties that are rare or even disfavored by natural selection (such as extreme thermostability in mesophilic organisms). These desirable functional optima may correspond to isolated high-fitness peaks separated by deep valleys in the evolutionary landscape (Figure 1c, d), representing trajectories that evolution is unlikely to traverse. Consequently, GMs trained solely on natural data tend to assign negligible probability to these regions, making them difficult to sample directly. Further biases arise from uneven phylogenetic representation and residual annotation errors in the underlying datasets (e.g. Refs. [22, 23, 24, 25]), which may further distort the learned landscape. In this sense, recent work highlights that simply increasing model scale does not guarantee monotonic improvements in fitness prediction [26].

However, when appropriately guided, GMs have the potential to access low-probability yet functionally optimal regions of protein space. More formally, the protein design objective can be defined as generating protein sequences *x* with desired properties *y*, where *y* may represent attributes such as thermostability, catalytic efficiency, binding specificity, or other design goals. By contrast, the primary training objective of a GM is to model the distribution of natural proteins, *p*(*x*). To bridge this gap, recent approaches aim to model the conditional distribution *p*(*x*|*y*), thereby biasing generation toward sequences with the desired attributes (Figure 1c). In many cases, this objective can also be viewed as an optimization problem, where the goal is to find *x* that maximizes *p*(*x*|*y*). Several recent techniques can be conceptually unified under this *p*(*x*|*y*) framework, showing strong success in aligning GMs with specific design objectives. In this review, we categorize these strategies into two broad classes: (i) parameter-updating alignment, which modifies the model parameters so that the learned distribution *p*(*x*) approximates *p*(*x*|*y*) and (ii) parameter-fixed steering, which guides generation toward target properties without altering the model’s underlying weights (Table 1).

### Parameter-updating alignment modifies weights to shift *p*(*x*)

Parameter-updating alignment modifies the underlying probability distribution by directly updating GMs’ parameters. The most straightforward approach in this category is supervised fine-tuning (SFT). In SFT, a pretrained GM is further optimized under the same objective used during pretraining but on a curated dataset of high-quality examples. For instance, a pretrained pLM can be fine-tuned on a carefully assembled dataset from a target enzyme family to generate novel and distant members of that family. This process adapts the model’s parameters to a specific domain, effectively shifting its generative prior to align with the target data. SFT has achieved notable success, including the generation of enzymes [27,28], gene editors [29,30], or bacteriophages [31].

**Table 1:** Overview of alignment and steering methods categorized by intervention stage within the generative process.

Category	Method	Intervention local	Description	Examples
Parameter-updating alignment	Supervised fine-tuning (SFT)	Model weights	Fit the model to well-curated data, shifting the learned distribution toward the data.	[27, 28, 29, 30]
	Reinforcement learning		Aligns the model on feedback data over model’s outputs (preference or reward-driven learning).	[21,32∗, 33∗, 34]
Parameter-fixed steering	Prompt and context programming	Prompting	Generation guided by structuring the input prompt with explicit instructions or templates.	[35, 36, 37, 38]
	Retrieval-augmented generation	Prompting	Enhances generation by dynamically incorporating external knowledge from a large corpus.	[39, 40∗∗, 41]
	Activation steering	Internal representation	Direct manipulation of hidden states (e.g. residual stream) to promote or suppress attributes.	[42, 43, 44]
	Output-dependent guidance	Decoding	Gradient-based guided generation in sequence space based on the inference output.	[10,45]
	Bayesian guidance	Decoding	Reweighs the probability distribution using Bayes’ theorem: $\tilde{p}\left(y|x\right)\propto p_{\theta }\left(y|x\right)\exp\left(\lambda s\left(y,x\right)\right)$.	[46]
	Sampling controls	Sampling	Alters sampling strategy (temperature, top-k, top-p, MCTS) to influence randomness.	[9,47,48]

MCTS, Monte Carlo Tree Search.

While SFT effectively specializes GMs toward generating samples representative of a particular dataset, it does not provide the model with the ability to discriminate by data quality, that is, to differentiate among varying degrees of a desired property. Similar limitations have been observed in natural language processing, where SFT alone often leads to suboptimal alignment with user intent and [33], in some cases, catastrophic forgetting [49]. Consequently, SFT is now commonly combined with reinforcement learning (RL) to achieve finer control over model behavior (e.g. Ref. [50]).

In RL, a model learns to make optimal decisions by interacting with an environment through trial and error, receiving feedback in the form of rewards or penalties to maximize its cumulative reward over time. Unlike SFT, in RL, the model is not provided with explicit examples of the desired outputs. Instead, the GM must infer and explore autonomously, potentially uncovering novel solutions that might not have been anticipated. More technically, a pretrained model is treated as a policy *π*_*θ*_(*x*) and updated to maximize a scalar reward or the probability over preferences while constraining excessive deviation from its pretrained distribution. Similarly to SFT, this process enables a transition from the unconditional distribution *p*(*x*) toward the desired conditional distribution *p*(*x*|*y*). Today, RL is central to the alignment of large language models (LLMs) and has driven remarkable advances across diverse fields, from autonomous driving to game playing.

While different RL algorithms for model alignment have been proposed over the years, we can broadly group them in two main categories, 1) deep RL–based approaches and 2) direct preference learning. In deep RL–based approaches, a reward model is first trained on scored responses based on desired properties *y* (e.g. human preferences). Subsequently, the reward model is used to update the policy (e.g. a pretrained LLM), typically via a policy gradient update, shifting the underlying policy distribution *p*(*x*) toward the properties *y*. REINVENT (2017) [51] provides an early attempt to apply deep RL–based approaches to the molecular field. REINVENT reframed the vanilla policy gradient update (i.e. REINFORCE) as an ‘augmented likelihood objective.’ This objective enables adjusting the generative model parameters to favor samples with high scores on a target property *y*, while retaining probability mass near the original distribution *p*(*x*), thereby preserving realism. Shortly after, Proximal Policy Approximation (PPO, 2017) [52] was introduced in the broader RL field, providing a key improvement over vanilla policy gradient methods; its clipped surrogate objective approximates a trust region, guaranteeing more stable policy updates.

The PPO update became the basis for RL from human feedback (RLHF) approaches [53], allowing to align an LLM to human preferences, while including a penalty for when the aligned model drifts too far from the original distribution *p*(*x*). The more recent Group Relative Policy Optimization (GRPO) algorithm (2024) [54] provides an alternative, where the reward is computed for a group of LLMs’ responses, augmenting the PPO update with a reward baseline (i.e. computed across the group), without needing to train any additional value model. Direct Preference Optimization (DPO, 2024) [55] introduced the second category of RL approaches for model alignment: direct preference learning. DPO leverages the same preference signal as PPO-based methods but casts alignment as a supervised objective over log-probability differences between ranked (human) preferences. In practice, this removes the need to train an explicit reward model, offering a simpler and potentially more stable route to preference alignment. These characteristics made DPO rapidly popular, and it has since spawned extensive variants of the original formulation.

These techniques have quickly met considerable success in the protein research realm, as summarized in Table 2. Deep RL–based approaches have been used to update pLMs with experimental measurements; for example, RL from experimental feedback aligns an ESM-based generator toward brighter CreiLOV variants while constraining drift from the original distribution [56]. In another example, Monte Carlo Tree Search (MCTS) guided by a policy–value network (AlphaZero-like [57]) has been applied to backbone construction, outperforming plain tree search on top-down design tasks [58]. In parallel, direct preference learning has been used to bias structure-conditioned models toward stability (preferring stabilizing over destabilizing sequences given a target backbone) to improve output quality within specific protein families and to reduce MHC-I epitope load while preserving the fold [21,32,59]. Finally, recent frameworks aim to unify and extend these ideas: mutation-policy RL methods propose on-policy sequence edits under task-specific oracles [56], while ProtRL generalizes the application of REINVENT, DPO, and GRPO algorithms to protein engineering with pLMs, exemplified on the design of low-nanomolar Epidermal Growth Factor Receptor (EGFR) inhibitors [33].

**Table 2:** Representative reinforcement learning frameworks applied to protein sequence design.

Method	Core objective	Protein design uses (2022–2025)
**REINFORCE** (vanilla policy gradient, 1992 [61])	Learns a policy such that actions with higher returns have higher likelihood of being sampled.	AB-gen [62]
**PPO** (Proximal Policy Optimization, 2017 [63])	Plain policy gradients such as REINFORCE are quite noisy and unstable. PPO clips the model’s gradient update to not deviate too much from original model distribution.	RLXF [56,64]
**AlphaZero MCTS** (Monte Carlo Tree Search with policy–value networks, 2018 [65])	A neural network with two heads is used to predict the most promising actions as well as the final rewards given a current position. This network is used to guide an MCTS to explore the most promising actions efficiently.	EvoPlay [66], HighPlay [67]
**DPO** (Direct Preference Optimization, [55])	Learns directly from ranked preference data, without requiring an explicit reward model.	ProteinDPO [32], ProtRL [33] and Park et al. [34]
**GRPO** (Group Relative Policy Optimization, [68])	For each prompt, it samples a group of G-candidate outputs, scores them with a reward, and uses relative (within-group) advantages to update the policy with a PPO-style clipped loss, without the need of a value model.	ProtRL [33], ProteinZero [64]

Summary of selected reinforcement learning (RL)–based approaches illustrating how core objectives have been adapted for protein or molecular design tasks between 2022 and 2025. The examples listed here are not exhaustive and focus on policy-based RL but highlight major methodological directions in the recent literature. For more details see Ref. [69], and Supplementary.

RLXF, Reinforcement Learning from eXperimental Feedback.

While direct preference learning approaches tend to be easier to work with than deep RL–based methods, it is still not clear which set of approaches lead to better performance under which conditions, and extensive benchmarking is needed [60], especially, in relation to protein design applications. Importantly, we believe the biggest limiting factor to the success of these methods in protein design is the difficulty of selecting good scoring metrics for most properties of interest.

### Parameter-fixed steering

**Figure 2:**
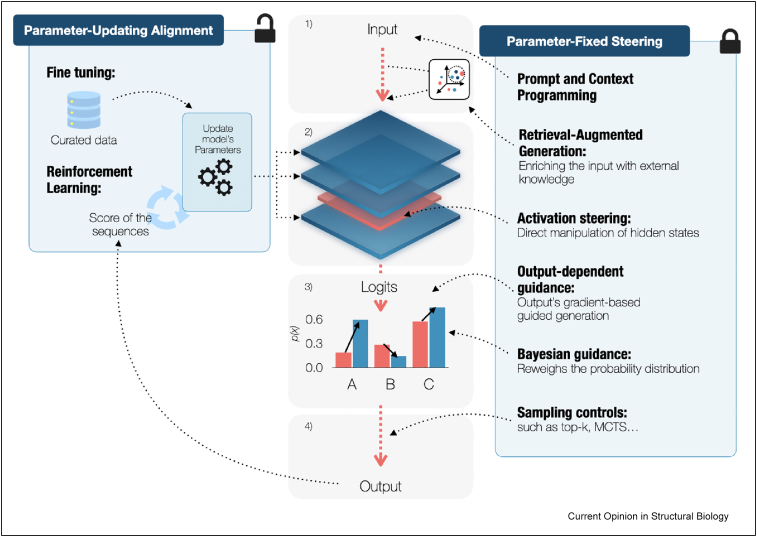
Different stages at which intervention can occur in protein generative modeling. Interventions may act on the model weights (parameter-updating alignment) or without changing the base model parameters (parameter-fixed steering). The latter category is further subdivided based on the stage of the model’s sampling pipeline where steering is applied: (1) specification of the input or context (e.g. prompting or retrieved context), (2) transformation into internal latent/activation representations, (3) decoding of latent representations into outputs, and (4) the sampling procedure that generates the final sequence or structure.

While parameter-updating alignment methods form a relatively well-defined class of methods, we use the term parameter-fixed steering to refer to a broader and more heterogeneous family of approaches. Despite their diversity, these methods share a common objective: conditioning model generation toward a desired property *y* without modifying the generative model’s base parameters. Conceptually, generation of learned GM can be decomposed into four stages: (1) an input or context specification stage (prompting), (2) the transformation of this input into internal latent or activation representations, and (3) the generation of the final output through the model’s decoding or (4) sampling procedure. The behavior of a GM can be influenced by intervening at any of these stages, as summarized in Figure 2.

#### Intervention at the input or context specification stage (prompting)

In the first stage, input conditioning specifies desired attributes through context or control tokens. When such attributes are provided during training, the model effectively learns conditional generation (sampling from an implicit *p*_*θ*_(*x*|*y*)) rather than *post hoc* steering of an unconditional *p*_*θ*_(*x*)). For example, several pLMs have been trained with various control tags, such as Enzyme Commission numbers (ZymCTRL, [27]), UniProt functional keywords (Progen1, [28]), taxonomy (Evo1/2, [8,9]), or combinations thereof [70].

If the conditioning signal is not explicitly provided during training, desired constraints can instead be imposed at inference time via partial specification or hard constraints. For example, structure-conditioned sequence design models can fix selected residues (e.g. functional motifs) while redesigning the remaining positions [36], and atomic-context conditioning can bias sequence design in the presence of ligands, nucleotides, or metals [37]. More explicitly, some sequence-generative models support infilling/inpainting, in which certain sequence segments are held fixed and the remaining residues are generated by masking and decoding the missing positions [35]. Others initialize sequences with unknown tokens (e.g. an all-X sequence) to induce structure hallucination in diffusion-based predictors, followed by iterative sequence redesign and structure reprediction [38].

Other approaches act directly on the latent encodings of protein sequences in a feedback optimization loop, exploring regions of protein-structure space that satisfy user-defined design objectives such as ColabDesign, BindCraft, and following methods [10,45]. It is important to note that in machine learning ‘generative’ strictly refers to models that sample from a learned probability distribution *p*(*x*). By this definition, techniques based on activation maximization, where a predictor is coupled with gradient-based optimization to produce sequences (e.g. BindCraft), are not generative in the formal sense as they do not define or sample from a prior. However, in the protein design community, these methods are often informally described as generative, and here, we include such approaches due to their prominent usage and success.

Alternatively, architectures such as BoltzGen [3] implement a conditional generative diffusion model with continuous guidance, in which encoded design conditions, such as binding-site specifications or structural constraints, are propagated throughout the denoising process to steer generation toward conformations consistent with the imposed design criteria [71]. With the aim to dynamically inject additional knowledge into the GM’s context, retrieval-augmented generation (RAG) has been successfully applied in different cases [39, 40∗∗, 41]. By drawing upon semantically related examples from large databases, RAG enables more informed sampling and can enrich the design process with functional or structural priors not explicitly encoded in the pretrained model. A recent example is Protriever [40], which introduces a retrieval-augmented pLM that jointly learns to retrieve homologous sequences and models their fitness, integrating evolutionary context at inference time without explicit structural supervision.

#### Intervention at the hidden states

A different approach acts directly within the hidden states of the network, targeting the second stage. Activation steering manipulates internal representations, often within the residual stream, by injecting vectors that correspond to interpretable latent directions. Sparse autoencoders (SAEs) have been used to identify such interpretable features in pLMs, revealing latent dimensions correlated with properties like enzymatic activity, hydrophobicity, or thermostability [42,43]. For example, Parsan et al. used SAE-derived features to bias structure predictions in ESMFold toward more hydrophobic conformations through feature steering [44], while Boxò et al. leveraged activity-associated features to steer ZymCTRL toward more active *α*-amylases [72].

#### Intervention at the output stage and sampling

Finally, control can also be applied at the level of output. Bayesian guidance reweighs the probability distribution encoded by GMs using Bayesian principles, effectively combining the model’s prior with external evidence or predictive scores. Such strategies have been applied in protein design, where sequence likelihoods are updated according to functional predictors or activity [46]. Similarly, sampling controls manipulate the stochasticity of the GM’s final output by, for example, manipulating ‘inference parameters’ like temperature, top-k, and top-p sampling, balancing diversity and fidelity, which is particularly important when sampling from vast sequence landscapes [48]. More advanced sampling techniques, such as beam search and MCTS, allow to consider multiple GMs’ inference trajectories, selecting the optimal ones (e.g. Refs. [9,47]).

Recent theoretical work underscores the generality of these approaches: flow matching in discrete state spaces has been shown to be equivalent to masked language modeling, autoregressive generation, and diffusion. This unifying perspective positions inference-time control as a suite of architecture-agnostic, plug-and-play techniques that can be ported across model classes with minimal modification [46].

### Conclusion and future prospects

Natural proteins are shaped by diverse and often competing forces: biochemical constraints, ecological pressure, and evolutionary mechanisms; making it difficult to define a single, global ‘fitness vector’ that can be captured by GMs. Combined with well-known dataset biases (e.g., see Refs. [22,23,25]), these properties impose severe limitations on how much we can leverage the natural distribution of proteins to design and engineer proteins *à la carte*, with full control over the design process.

Over the last two years, we have witnessed the emergence of several powerful methods for guiding GMs toward desired regions of the learned protein distribution (i.e. those encoding target properties for engineering). Here, we describe these methods and classify them into two broad categories: parameter-updating alignment and parameter-fixed steering. These strategies have already been used to bias protein generative models toward brighter fluorescent proteins (e.g. CreiLOV variants) [56], increased stability [32], reduced MHC-I epitope load [59], and improved target-binding designs, such as low-nanomolar EGFR inhibitors [33].

Despite this progress, several challenges still limit the immediate applicability of these approaches to fully autonomous protein engineering. A recurring issue across both categories is poor out-of-distribution generalization. Parameter-fixed steering relies on the assumption that the pretrained model already encodes meaningful structure in the relevant regions of sequence space; consequently, steering cannot readily extrapolate beyond representations the base model has not learned. Parameter-updating strategies offer greater flexibility by reshaping the learned distribution through fine-tuning, potentially extending coverage beyond pretraining. However, this flexibility comes at the cost of increased dependence on downstream data quality and optimization signals.

Many curated datasets used for SFT, as well as *in silico* scoring functions used in RL, remain rooted in the natural protein distribution and therefore inherit the same evolutionary biases and constraints that we ultimately attempt to overcome (e.g. Refs. [21,36,73]). In this context, lab-in-the-loop frameworks that iteratively combine generative models with experimental validation represent a particularly promising direction as they could progressively enrich the data distribution [74]. Complementary efforts aim to improve pretraining and optimization data by diversifying sequence sampling across the tree of life [74] or introducing inductive biases that leverage evolutionary context through Multiple Sequence Alignments, enabling more efficient training [75] and sampling [25].

A second major bottleneck concerns the availability of accurate and reliable scoring metrics. For many properties of interest—such as catalytic activity—robust predictive oracles remain difficult to obtain. Incorporating physics-based scoring methods, such as Rosetta [76] and FoldX [77], into RL pipelines may help ground optimization in physically informed principles and reduce reliance on purely data-driven signals.

More fundamentally, it remains unclear whether alignment and steering methods genuinely uncover novel functional solutions or primarily recombine patterns already present in the training data. Addressing this question will require systematic and controlled empirical comparisons across diverse tasks and evaluation settings, allowing the field to rigorously assess the relative strengths and limitations of parameter-updating and parameter-fixed approaches [60,78]. As in many areas of biology, different strategies may prove optimal in different regimes—for example, *de novo* generation versus sequence optimization. Ultimately, advancing beyond the natural sequence distribution while maintaining reliability will be the defining challenge of the next phase of protein generative modeling, shaping the path toward truly controllable and new-to-nature protein design.

## Declaration of competing interest

The authors declare that they have no known competing financial interests or personal relationships that could have appeared to influence the work reported in this paper.

## Figures and Tables

**Table 3:** Summary of works reported in this manuscript, with links to code repositories, when available.

Title	GitHub	Reference	Type	GM
Continued on next page				
Conditional language models enable the efficient design of proficient enzymes	–	[27]	Fine-tuning	pLM
Large language models generate functional protein sequences across diverse families	salesforce/progen	[28]	Fine-tuning	pLM
Design of highly functional genome editors by modeling the universe of CRISPR-Cas sequences	Profluent-AI/OpenCRISPR	[29]	Fine-tuning	pLM
Discovery and protein language model-guided design of hyperactive transposases	Integra-tx/Piggybac_bioprospecting_pipeline	[30]	Fine-tuning	pLM
Aligning protein generative models with experimental fitness via Direct Preference Optimization	evo-design/protein-dpo	[32]	Reinforcement learning (DPO)	pLM
Guiding Generative Protein Language Models with Reinforcement Learning	AI4PDLab/ProtRL	[33]	Reinforcement learning (DPO, GRPO)	pLM
Improving Protein Sequence Design through Designability Preference Optimization	–	[34]	Reinforcement learning (DPO)	Diffusion
Simulating 500 million years of evolution with a language model	evolutionaryscale/esm	[21]	Fine-tuning	pLM
Protein generation with evolutionary diffusion: sequence is all you need	microsoft/evodiff	[35]	Prompt and context programming	Diffusion
Robust deep learning–based protein sequence design using ProteinMPNN	dauparas/ProteinMPNN	[36]	Prompt and context programming	–
Atomic context-conditioned protein sequence design using LigandMPNN	dauparas/LigandMPNN	[37]	Prompt and context programming	–
Protein Hunter: exploiting structure hallucination within diffusion for protein design	yehlincho/Protein-Hunter	[38]	Prompt and context programming	–
PoET: A generative model of protein families as sequences-of-sequences	OpenProteinAI/PoET	[39]	Retrieval-augmented generation	pLM
Protriever: End-to-End Differentiable Protein Homology Search for Fitness Prediction	–	[40]	Retrieval-augmented generation	pLM
Understanding protein function with a multimodal retrieval-augmented foundation model	OpenProteinAI/PoET-2	[41]	Retrieval-augmented generation	pLM
Mechanistic Interpretability of Antibody Language Models Using SAEs	–	[42]	Sparse autoencoders	pLM
Interpreting and steering protein language models through sparse autoencoders	edithvillegas/plm-sae	[43]	Sparse autoencoders	pLM
Towards Interpretable Protein Structure Prediction with Sparse Autoencoders	–	[44]	Sparse autoencoders	–
One-shot design of functional protein binders with BindCraft	martinpacesa/BindCraft	[10]	Output-dependent guidance	–
Boltzdesign1: Inverting All-Atom Structure Prediction Model for Generalized Biomolecular Binder Design	yehlincho/BoltzDesign1	[45]	Output-dependent guidance	Diffusion
ProteinGuide: On-the-fly property guidance for protein sequence generative models	–	[46]	Bayesian guidance	Diffusion, pLM
Genome modeling and design across all domains of life with Evo 2	arcinstitute/evo2	[9]	Fine-tuning	gLM
Sampling Protein Language Models for Functional Protein Design	i3LBI19-OATML/sampling_plm	[47]	Sampling controls	pLM
ProtGPT2 is a deep unsupervised language model for protein design	https://huggingface.co/nferruz/ProtGPT2	[48]	Fine-tuning	pLM
Ab-Gen: Antibody Library Design with Generative Pre-trained Transformer and Deep Reinforcement Learning	https://zenodo.org/records/7657016	[79]	Reinforcement learning	pLM
BoltzGen: Toward universal binder design	HannesStark/boltzgen	[3]	Output-dependent guidance	Diffusion
CMaDiff: Cross-modal aligned diffusion for controllable protein generation	HPC-NEAU/PhysChemDiff	[71]	Reinforcement learning	Diffusion
Self-play reinforcement learning guides protein engineering (EvoPlay)	melobio/EvoPlay	[66]	Reinforcement learning	Policy/Reinforcement learning
HighPlay: Cyclic peptide sequence design based on reinforcement learning and protein structure prediction	–	[67]	Reinforcement learning	Reinforcement learning + structure predictor
ProteinZero: Self-improving protein generation via online reinforcement learning	–	[64]	Reinforcement learning	pLM
Functional alignment of protein language models via reinforcement learning (RLXF)	RomeroLab/RLXF	[56]	Reinforcement learning	pLM

DPO, Direct Preference Optimization; GM, generative model; GRPO, Group Relative Policy Optimization; pLM, protein language model; RLXF, Reinforcement Learning from eXperimental Feedback; SAE, sparse autoencoder.

CRISPR, Clustered Regularly Interspaced Short Palindromic Repeats.

## Appendix A Supplementary

### Reinforcement learning losses (Supplementary to Table 2)

This section details the mathematical formulations of the reinforcement learning (RL) objectives summarized in Table 2. Here *π*_*θ*_ denotes the policy, while the *D*_*KL*_(*π*_*θ*_‖*π*_*ref*_) is a Kullback-Leibler (KL) regularization term to penalize strong deviations from the reference model *π*_*ref*_.

#### REINFORCE.

The REINFORCE algorithm maximizes the expected reward by increasing the log-likelihood of sampled actions by their associated return *r*(*x*). The corresponding loss is expressed as follows:

(1) $$L_{\text{REINFORCE}}=-E_{x∼\pi_{\theta }}\left[r\left(x\right)\;\log\pi_{\theta }\left(x\right)\right]$$

#### Proximal Policy Optimization.

PPO stabilizes training by preventing excessively large policy updates that may cause divergence. This is achieved by clipping the policy-ratio term *r*_*i*_(*θ*) = *π*_*θ*_(*a*_*i*_|*s*_*i*_)/*π*_ref_(*a*_*i*_|*s*_*i*_). The clipped objective is as expressed follows:

(2) $$L_{\text{PPO}}=-\frac{1}{G}\overset{G}{\underset{i=1}{\sum }}\left[\min\left(r_{t}\left(\theta \right)\;{\hat{A}}_{t},\;clip\left(r_{t}\left(\theta \right),1-\epsilon ,1+\epsilon \right)\;{\hat{A}}_{t}\right)\right]$$

where ${\hat{A}}_{t}$ denotes the advantage and *ϵ* defines the clipping range. While the advantage can be computed in different ways (e.g. Group Relative Policy Optimization [GRPO] style), in PPO, it is classically computed relative to a learned value function predicted the per-token expected reward. In RL from human feedback, a KL penalty relative to a fixed reference model is added to the reward, ensuring that the new policy remains close to this reference.

#### AlphaZero.

This trains a single neural network with two heads, one that predicts the probability distribution over actions *p*_*θ*_(*a*|*s*) and the other that predicts the expected sum of rewards given a state s *v*_*θ*_(*s*). The training data come from *self-play* using a Monte Carlo Tree Search (MCTS) that produces the final real outcome *z* and the used policies *π* (i.e. visit counts distribution for MCTS rollouts). The training loss is expressed in the following:

(3) $$L\left(\theta \right)={\left(z-v_{\theta }\left(s\right)\right)}^{2}-\underset{a}{\sum }\pi \left(a|s\right)\log p_{\theta }\left(a|s\right)+c\|\theta {\|}_{2}^{2}$$

where ${\left(z-v_{\theta }\left(s\right)\right)}^{2}$ is the value loss (mean square error between predicted and true value), −*∑*_*a*_*π*(*a*|*s*) log  *p*_*θ*_(*a*|*s*) is the policy loss (cross-entropy between MCTS policy and the network policy and $c\|\theta {\|}_{2}^{2}$ is a regularization term to avoid large weights updates (scaled by a constant *c*).

#### Direct Preference Optimization

Introduced by Rafailov et al. (2023), Direct Preference Optimization (DPO) learns the optimal policy directly from pairwise preference data without requiring a reward model or value function. Given preferred (*x*_*w*_) and dispreferred (*x*_*l*_) samples, the objective is as follows:

(4) $$L_{\text{DPO}}=-E_{\left(x_{w},x_{l}\right)}\left[\log\;\sigma \left(\beta \left[\log\pi_{\theta }\left(x_{w}\right)-\log\pi_{\theta }\left(x_{l}\right)\right]\right)\right],$$

where *σ* is the sigmoid function and *β* controls the strength of preference separation.

#### Group Relative Policy Optimization

GRPO extends PPO by organizing responses into |*o*| groups and computing advantages relative to each group’s mean performance, thereby reducing variance of each update and improving stability. It does not require a separate value model. The objective is as follows:

(5) $$L_{\text{GRPO}}=-\frac{1}{G}\overset{G}{\underset{i=1}{\sum }}\frac{1}{\left|o_{i}\right|}\overset{\left|o_{i}\right|}{\underset{t=1}{\sum }}\min\left(r_{i,t}\left(\theta \right)\;{\hat{A}}_{i,t},\;clip\left(r_{i,t}\left(\theta \right),1-\epsilon ,1+\epsilon \right)\;{\hat{A}}_{i,t}\right)+\beta \;D_{\text{KL}}\left(\pi_{\theta }\|\pi_{\text{ref}}\right)$$

where each group *o*_*i*_ contains trajectories with similar characteristics. ${\hat{A}}_{i,t}$ denotes the group-relative advantage, which is computed as follows:

(6) $${\hat{A}}_{i,t}=\frac{r_{i}-{\text{mean}}_{t}\left(r\right)}{{\text{std}}_{t}\left(r\right)}$$

with the mean_*t*_ and std_*t*_ being computed over the group response.

### 2.2 Retrieval-augmented generation

Retrieval-augmented generation augments a parametric policy *π*_*θ*_ with external knowledge retrieved from a database D. Given an input *x*, a retriever *p*_*η*_(*d*|*x*) selects top-k relevant elements *d* in the dataset D and marginalize the generator *π*_*θ*_.

(7) $$p_{\theta }\left(y|x\right)=\underset{d\in D}{\sum }p_{\theta }\left(y|x,d\right)\;p_{\eta }\left(d|x\right)$$

This formulation enables the model to combine parametric knowledge (stored in *θ*) with nonparametric memory (stored in D), improving factual grounding. In protein modeling settings, the database may consist of known sequences, structures, or functional annotations.

### 2.3 Monte Carlo Tree Search

MCTS is a planning algorithm that builds a search tree by iteratively simulating trajectories and updating value estimates.

At each state *s* (e.g. a partially generated sequence), MCTS maintains a visit count *N*(*s*), action visit counts *N*(*s*, *a*), and action-value estimates *Q*(*s*, *a*). During selection, actions are chosen using an upper confidence bound criterion:

(8) $$a^{*}=\arg\max_{a}\left[Q\left(s,a\right)+c\sqrt{\frac{\log\;N\left(s\right)}{N\left(s,a\right)+1}}\right],$$

where *c* controls the exploration–exploitation trade-off.

After expanding a new node, a rollout (simulation) provides a reward *r*, which is backpropagated to update:

(9) N(s)←N(s)+1,

(10) N(s,a)←N(s,a)+1,

(11) $$Q\left(s,a\right)\leftarrow \frac{N\left(s,a\right)\;Q\left(s,a\right)+r}{N\left(s,a\right)+1}.$$

When combined with policy–value networks (as in AlphaZero-style approaches), a learned prior *p*_*θ*_(*a*|*s*) can guide exploration, improving search efficiency in large combinatorial spaces such as protein sequence design.

### 2.4 Sparse autoencoders

Sparse Autoencoders (SAEs) are unsupervised models designed to learn compressed and interpretable latent representations. An encoder *f*_*θ*_ maps an input *x* to a latent representation *z*, and a decoder *g*_*ϕ*_ reconstructs the input into $\hat{x}$. Encoder and decoder are trained with a reconstruction loss, which is usually the mean squared error:

(12) $$L_{\text{rec}}=\|x-\hat{x}{\|}_{2}^{2}$$

To encourage sparsity in the latent representation, a regularization term is added, typically an *ℓ*_1_ penalty:

(13) $$L_{\text{SAE}}=L_{\mathrm{rec}}+\lambda \;L_{\mathrm{aux}}$$

where *λ* controls the sparsity strength. SAEs are commonly used for representation disentanglement, feature discovery, and interpretability analysis in large language and protein models. Ideally, SAE features are *atomic*, meaning each latent dimension corresponds to a single, minimal, and semantically coherent factor (rather than an entangled mixture of multiple concepts), so features can be interpreted and recombined predictably.
